# Germline Specific Expression of a *vasa* Homologue Gene in the Viviparous Fish Black Rockfish (*Sebastes schlegelii*) and Functional Analysis of the *vasa* 3**′** Untranslated Region

**DOI:** 10.3389/fcell.2020.575788

**Published:** 2020-10-28

**Authors:** Li Zhou, Xueying Wang, Shuran Du, Yanfeng Wang, Haixia Zhao, Tengfei Du, Jiachen Yu, Lele Wu, Zongcheng Song, Qinghua Liu, Jun Li

**Affiliations:** ^1^The Key Laboratory of Experimental Marine Biology, Center for Ocean Mega-Science, Institute of Oceanology, Chinese Academy of Sciences, Qingdao, China; ^2^Laboratory for Marine Biology and Biotechnology, Qingdao National Laboratory for Marine Science and Technology, Qingdao, China; ^3^University of Chinese Academy of Sciences, Beijing, China; ^4^Weihai Shenghang Aquatic Product Science and Technology Co., Ltd., Weihai, China

**Keywords:** *vasa*, *Sebastes schlegelii*, gonad, germ cell, development, 3cpsdummy′UTR, localization

## Abstract

Germ cells play a key role in gonad development. As precursors, primordial germ cells (PGCs) are particularly important for germline formation. However, the origination and migration patterns of PGCs are poorly studied in marine fish, especially for viviparous economic species. The *vasa* gene has been widely used as a germ cell marker to identify a germline because *vasa* RNA is a component of germ plasm. In this study, we described the expression pattern of black rockfish (*Sebastes schlegelii*) *vasa* (*Ssvas*) in gonadal formation and development by *in situ* hybridization. The results showed that *Ssvas* failed in localization at the cleavage furrows until the late gastrula stage, when PGCs appeared and migrated to the genital ridge and formed elongated gonadal primordia at 10 days after birth. This study firstly revealed the PGCs origination and migration characteristics in viviparous marine fish. Furthermore, we microinjected chimeric mRNA containing EGFP and the 3′untranslated region (3′UTR) of *Ssvas* into zebrafish (*Danio rerio*) and marine medaka (*Oryzias melastigma*) fertilized eggs for tracing PGCs. We found that, although *Sebastes schlegelii* lacked early localization, similar to red seabream (*Pagrus major*) and marine medaka, only the 3′UTR of *Ssvas vasa* 3′UTR of black rockfish was able to label both zebrafish and marine medaka PGCs. In comparison with other three Euteleostei species, besides some basal motifs, black rockfish had three specific motifs of M10, M12, and M19 just presented in zebrafish, which might play an important role in labeling zebrafish PGCs. These results will promote germ cell manipulation technology development and facilitate artificial reproduction regulation in aquaculture.

## Introduction

As highly specialized cells, germ cells play a key role in germline development (Xu et al., 2010). In fish, the germ cells undergo a series of basic biological processes, including formation, migration, proliferation, and differentiation, and eventually develop into mature gametes (Braat et al., 1999; Nishimura and Tanaka, 2014). Healthy gonad development and gametogenesis in fish are important for aquaculture. Therefore, investigating the whole development process of germ cells facilitates the reproduction regulation in aquaculture, such as sex control, gonad maturation induction, and artificial insemination (Piferrer, 2001). At the same time, it promotes germ cell manipulations, in which the time of gonad formation can provide a reference for germ cell transplantation (Takeuchi et al., 2004; Okutsu et al., 2006).

Primordial germ cells (PGCs), the precursors of germ cells, are set aside from somatic cells early in embryogenesis (Goto-Kazeto et al., 2010). PGCs arrive in the genital ridge through a long-range cell migration, and they are covered by somatic cells to form elongated gonadal primordia. In recent years, many maternal-effect genes such as *vasa*, *dnd*, *dazl*, and *nanos* localized in germplasm have been identified and extensively used as germ cell markers to study origination and migration of PGCs in fish (Xu et al., 2007; Lindeman and Pelegri, 2010; Yoshizaki et al., 2010; Hong et al., 2016). This reveals that the PGC migration routes and gonadal formation times vary from fish species. So far, there are two classical origination and migration patterns of PGCs. One representative is the zebrafish (*Danio rerio*), in which germ plasm granules in fertilized eggs are concentrated to the distal parts of the cleavage furrows, resulting in four aggregates during the first two cleavage divisions, and these cluster cells will differentiate into PGCs at the late blastula (Yoon et al., 1997). The other representative is the medaka (*Oryzias latipes*), whose granules are distributed uniformly throughout each blastomere until the onset of epiboly, and the PGCs start to appear at late gastrulation, lacking early localization (Shinomiya et al., 2000). Once PGCs form, they migrate and concentrate on the dorsal peritoneum, under the notochord at the hatching stage. The PGCs undergo a post-hatching migration in most oviparous fish. According to histological studies, PGCs migrate from the upper part of the body cavity to the rear and settle on the dorsal side of the gut, a location that future gonad forms. The settlement time of PGCs is species specific. In ukigori (*Gymnogobius urotaenia*), PGCs reached the genital ridge at 6–8 days post-hatching (dph) (Saito et al., 2004), while in turbot (*Schophthalmus maximus*), Japanese flounder (*Paralichthys olivaceus*), and yellowtail kingfish (*Seriola lalandi*), PGCs arrived in the gonad formation location at 15–20 dph (Fernandez et al., 2015; Zhao et al., 2017; Yang et al., 2018). However, the time of primordial gonadal formation of viviparous fish has not been reported so far. After migration, the germ cells begin mitosis and meiosis, and the formed gonads differentiate into presumptive ovary or testis (Gao et al., 2009). Under the control of the endocrine regulation and the external environment, gonads gradually develop to maturity and produce sexual gametes.

The *vasa* is the first germ gene identified in fish, which encodes an ATP-dependent RNA helicase of the DEAD (Asp–Glu–Ala–Asp)-box family (Knaut et al., 2000). It has been proved to be able to fulfill potential roles in germ cell origination and migration and maintain throughout the development of germ cells in fish (Kobayashi et al., 2000; Lin et al., 2012b; Ye et al., 2016). Moreover, the function of *vasa* varied considerably in diverse species. In medaka, *vasa* knockdown led to defects in PGCs migration but not proliferation, motility, identity, and survival (Li et al., 2009), while in zebrafish, disruption of *vasa* resulted in sterility in males (Hartung et al., 2014). As a universal germ cell marker, the *vasa* gene was used to identify the fish germline in economic species such as rainbow trout (*Oncorhynchus mykiss*) (Yoshizaki et al., 2010), Dabry’s sturgeon (*Acipenser dabryanus*) (Ye et al., 2016), and Atlantic cod (*Gadus morhua*) (Presslauer et al., 2012). The *vasa* mRNA has been shown to degrade rapidly in somatic cells but remain stable in PGCs, which are all mediated by the 3′untranslated region (3′UTR) (Knaut et al., 2002; Wolke et al., 2002; Mishima et al., 2006). For the inherent nature of *vasa* 3′UTR, the chimeric mRNA fused fluorescent protein-coding region to 3′UTR could be microinjected into fertilized eggs to trace PGCs (Kataoka et al., 2006; Ye et al., 2016). This is a highly efficient, fast, and specific non-transgenic method for labeling PGCs *in vivo* and has been used in a variety of fish species (Yoshizaki et al., 2005; Lin et al., 2012a; Zhou et al., 2019). Moreover, the chimeric mRNA from one species not only labels itself but also can successfully trace other species, even the genetic highly divergent species. For example, the chimeric mRNA (GFP-*Pmvas* 3′UTR) that contained GFP and the 3′UTR of red seabream (*Pagrus major*) could be used to trace migration of PGCs in medaka (Lin et al., 2012a). However, chimeric mRNA from one species cannot label PGCs from all species, just as the red seabream *vasa* 3′UTR failed to trace turbot PGCs, although they could label the medaka (Lin et al., 2012a; Zhou et al., 2019). This indicated that the conserved localization mechanism of *vasa* 3′UTR may relate to specific functional elements (Knaut et al., 2002; Kataoka et al., 2006; Zhou et al., 2019). For labeling ability of *vasa* 3′UTR, there may be rules to follow. However, the relevant researches in other species are limited, so it is still unclear.

Black rockfish (*Sebastes schlegelii*) is one of the most important economic rockfishes in the western North Pacific and inhabits the coastal waters of China, Japan, and Korea (Kai and Soes, 2009). As a viviparous fish, the embryos develop in female cavity for 1–2 months until birth (Liu et al., 2019). In previous research, *Sebastes schlegelii vasa* (*Ssvas*) was only detected and highly expressed in gonad during the reproductive cycle (Mu et al., 2013). There is still a lack of detailed research on germ cells during gonadal formation and development in this viviparous fish species. In this study, we aimed to describe the origination and migration pattern of *Sebastes schlegelii* PGCs in gonad formation process by *vasa* and explored the relationship between *vasa* RNA early localization and species evolution. Furthermore, we also identified the germ cells development and maturation process with *vasa*, verified the labeling ability of *Ssvas* 3′UTR in zebrafish and marine medaka (*Oryzias melastigma*), and analyzed the conservative motifs of *vasa* 3′UTR in comparison with several other species.

## Materials and Methods

### Fish and Samples

Matured black rockfish used in this study were obtained from Penglai, China. Ten sexually mature individuals in November were anesthetized with a 0.05% solution of 3-aminobenzoic acid ethyl ester methanesulfonate-222 (MS-222) (Sigma–Aldrich, United States). The tissues of ovary and testis were rapidly removed. Some samples were immediately immersed in liquid nitrogen for cDNA synthesis, while others were fixed overnight with 4% paraformaldehyde in phosphate-buffered saline (PBS) and then preserved in 70% ethanol for section *in situ* hybridization (SISH). Twenty sexually mature females in April to May were also anesthetized with MS-222. Moreover, the embryos of different development stages in the ovary were removed and fixed by 4% paraformaldehyde in PBS overnight and stored at −20°C in PBS with 50% formamide for whole mount *in situ* hybridization (WISH).

One-year-old and hatching fry of black rockfish were reared at Shenghang Sci-Tech Co., Ltd. (Shandong Province, China). The testes and ovaries of six 1-year-old individuals were removed after anesthesia and fixed in 4% paraformaldehyde as above. Starting from hatching, about 20 hatched fry were fixed with 4% paraformaldehyde every day for 1 month. After 1 day of fixation, the gonadal tissue samples and fry were stored at 70% ethanol for SISH.

All experiments were performed in accordance with the relevant national and international guidelines and approved by the Institutional Animal Care and Use Committee, Institute of Oceanology, Chinese Academy of Sciences.

### Sequence Analyses

A homology search of the amino acid sequence of *Ssvas* was carried out using the National Center for Biotechnology Information website^1^. The amino acid sequences were aligned using the AlignX program in Vector NTI, Suite 8 software package (Life Technologies). Phylogenetic analysis was conducted with Mega4 software using the neighbor-joining method, and the bootstrap replicates were set to 1,000 (Saitou and Nei, 1987). Some potential regulatory motifs in the 3′UTRs of teleost *vasa* were searched by MEME motif analysis (Bailey and Elkan, 1994).

### *In situ* Hybridization

Total RNA was extracted from the ovaries of *Sebastes schlegelii* using RNA fast 200 (Fastagen, China). In accordance with the manufacturer’s instructions, the first-strand cDNA was synthesized by the PrimeScript^TM^ RT reagent kit with gDNA Eraser (Takara, Japan). Then, the cDNA was stored at −20°C as a probe synthesis template.

*vasa* mRNA distribution was detected in embryo and tissue by *in situ* hybridization (ISH), and the protocols followed the methods described previously (Lin et al., 2012b), with a few modifications as detailed below. Briefly, the 857 bp *Ssvas* fragment (nucleotides 1,540–2,396 bp; accession no. JN634874) was inserted into pGEM-T Easy vector (Promega, Madison, WI). The primers are shown in Table 1. Sense and antisense RNA probes were synthesized by *in vitro* transcription from a vector under the drive of the T7 or SP6 promoter with the DIG RNA Labeling Kit (Roche, Mannheim, Germany). The RNA probes were treated with RNase-free water and purified with SigmaSpin^TM^ Sequencing Reaction Clean-Up (Sigma–Aldrich). ISH was conducted by chemical stain with BCIP/NBT substrates on whole mount and sections samples.

**TABLE 1:** Primers used in the study.

Primer	Sequence	Purpose
BrvasF	CTGATTTCATTGCCACTT	ISH probe for detecting *Ssvas* mRNA
BrvasR	ATTGGTGACCTTTATGGA	

BrutrF	AGGAAATATTAGAGCACA	Obtaining black rockfish and zebrafish *vasa* 3′UTRs by RT-PCR
BrutrR	GCACAAATACTTATTTAT	
ZbutrF	CTGGCCTCACACCTGTTA	
ZbutrR	CACCAGTATCCGTCTTTATT	

VeBrF	GAGCTGTACAAGTAAAGGAA ATATTAGAGCACA	Obtaining *vasa* 3′UTR fragments for Gibson assembly
VeBrR	GAATTCACTAGTGATGCACAA ATACTTATTTAT	
VeZbF	GAGCTGTACAAGTAACTGGC CTCACACCTGTTA	
VeZbR	ATTTCTGCCTATGACCACTA GCTTAAGGGCGCC	

BrvecF	ATCACTAGTGAATTCGCG	Obtaining vector fragments for Gibson assembly
BrvecR	TTACTTGTACAGCTCGTC	
ZbvecF	ATCGAATTCCCGCGGCCG	
ZbvecR	CTGCTCGACATGTTCATT	

T7	TAATACGACTCACTATAGGG	Preparation synthesis templates for chimeric mRNAs
BrutrR	GCACAAATACTTATTTAT	
SP6	ATTTAGGTGACACTATAG	
ZbutrR	CACCAGTATCCGTCTTTATT	

For WISH, embryos were dechorionated, and dehydrated and rehydrated through a graded series of methanol after being taken out from 50% formamide in PBS. The embryos were digested with proteinase K (10 μg/ml) (5–10 min for embryo < 24 h; 15–30 min for embryo > 24 h) and refixed before hybridization. For SISH, samples were dehydrated through a graded series of ethanol, embedded in paraffin wax, and cut into 5 μm-thick sections for testis and 7 μm for ovary. The sections were de-waxed using two washes of xylene and rehydrated through an ethanol gradient. After being washed with PBST, they were refixed using 4% paraformaldehyde in PBS and digested with proteinase K (10 μg/ml) for 10 min. The WISH and SISH were performed with the probes at 65°C for 14 h. Stained samples were observed and photographed through a Nikon ENi microscope, using a Nikon DS-Ri2 imaging system.

### Prepare of EGFP-*Ssvasa* 3′UTR and mCherry-*Drvasa* 3′UTR mRNAs

Chimeric mRNAs which fused EGFP or mCherry to the 3′UTR of *Sebastes schlegelii* or *Danio rerio vasa* gene were synthesized by *in vitro* transcription, and the protocols followed the methods described previously (Zhou et al., 2019). The main process is as follows: firstly, template plasmids were constructed using Gibson assembly, and the 3′UTRs of EGFP*-Pmvasa* 3′UTR and mCherry-*Omvasa* 3′UTR plasmids in our lab were replaced by *vasa* 3′UTRs of black rockfish and zebrafish, respectively. Then, according to the plasmids, the transcription templates were synthesized by PCR. After purification, capped chimeric mRNAs were synthesized using mMESSAGE mMACHINE SP6/T7 kit (Ambion, United States). The primers for preparing mRNAs are also shown in Table 1. In the previous study, the EGFP*-Pmvasa* 3′UTR and mCherry-*Omvasa* 3′UTR mRNAs that contained red seabream and marine medaka *vasa* 3′UTRs have been synthesized in our lab (Zhou et al., 2019).

### Microinjection and Observation

Zebrafish and marine medaka were maintained at 28 ± 0.5°C (16 h light/8 h dark) at the fish facility of the Institute of Oceanology, Chinese Academy of Sciences. The marine medaka is a truly euryhaline species closely related to the medaka. Embryos were collected within 30 min after fertilization. To verify the labeling ability of *Ssvas* 3′UTR in zebrafish and marine medaka, the EGFP-*Ssvasa* 3′UTR and mCherry-*Drvasa* 3′UTR mRNAs were co-injected into zebrafish embryos at the 1–2 cell stage, and the EGFP-*Ssvasa* 3′UTR and mCherry-*Omvasa* 3′UTR mRNAs were co-injected into marine medaka embryos in the same manner. Similarly, to compare the ability of *vasa* 3′UTRs among *Sebastes schlegelii* and other teleost in marking zebrafish PGCs, the EGFP*-Pmvasa* 3′UTR and mCherry-*Omvasa* 3′UTR mRNAs were also microinjected into zebrafish embryos as above. Microinjected eggs were cultured in a biochemical incubator with 28°C for EGFP or mCherry observation and photography under fluorescent microscope (Nikon ENi, Japan) at different stages. The microscope equipped with a FITC filter or a TRITC filter and a DS-Ri2 imaging system.

## Results

### Structure and Phylogenetic Analysis of *Sebastes schlegelii* Vasa Protein

**FIGURE 1:**
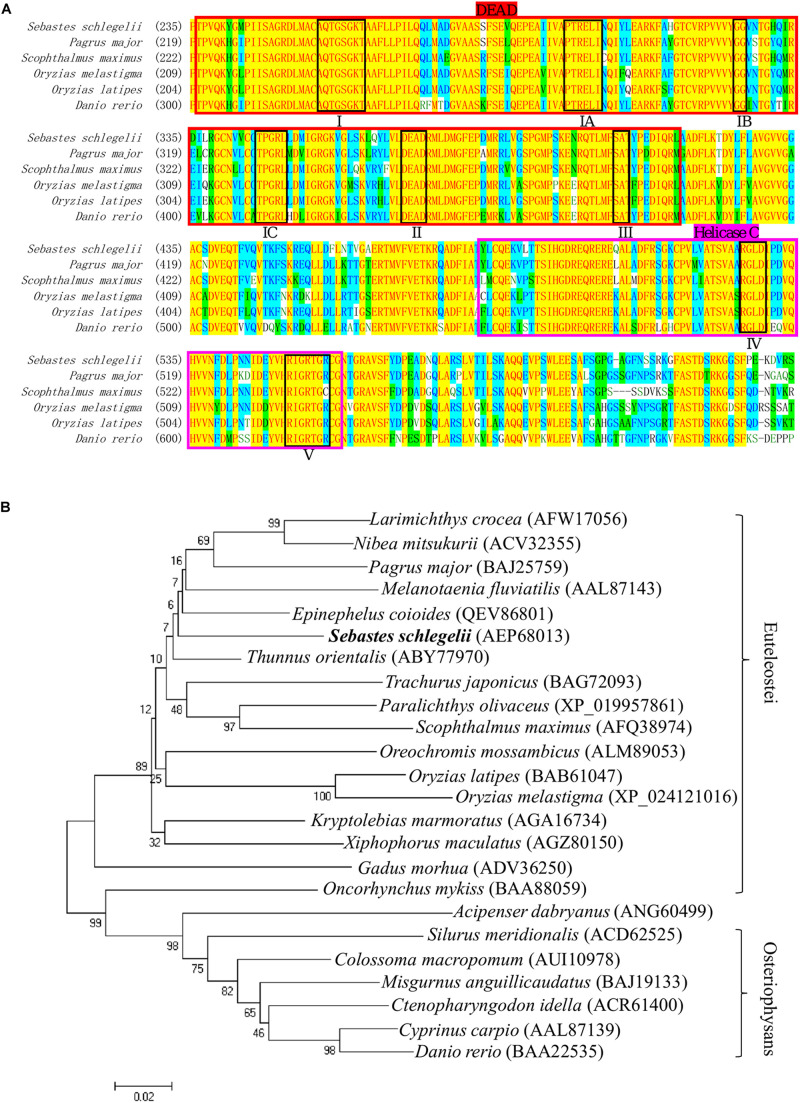
Multiple alignment and phylogenetic analysis of the Vasa between *Sebastes schlegelii* and other species. **(A)** Multiple alignment was carried out using the AlignX program. The eight conserved motifs are shown in block, including I domain (AQTGSGKT), IA domain (PTREL), IB domain (GG), IC domain (TPGRL), II domain (DEAD), III domain (SAT), IV domain (ARGLD), and V domain (GRTGR). The DEAD-box sequence was indicated in bright red, while Helicase C was in rose red. **(B)** The phylogenetic tree of Vasa was established by MEGA4 with neighbor-joining method. The numbers adjacent to nodes indicated bootstrap percentage value for 1000 replicates.

The full-length cDNA of *Ssvas* had been isolated in the previous research. In this study, multiple alignment of different fish Vasa proteins showed that there were eight consensus motifs (I, IA, IB, IC, II, III, IV, V) in the DEAD-box protein family (Figure 1A). This also revealed that the DEAD domain was composed of 180 amino acids and the superfamily Helicase C-terminal (Helicase C) of 77 amino acids, which had high similarity in teleost (Figure 1A). The *Sebastes schlegelii* presented Vasa typical conserved domains (Figure 1A). Phylogenetic analysis of Vasa protein exhibited that *Sebastes schlegelii* belonged to Euteleostei species (Figure 1B). In phylogeny, compared to Osteriophysans species like *Danio rerio*, *Sebastes schlegelii* was closer to some Euteleostei fish, such as *Thunnus orientalis*, *Scophthalmus maximus*, *Pagrus major*, *Oryzias latipes*, and *Oncorhynchus mykiss* (Figure 1B).

### Spatial Localization of *Ssvas* RNA During Embryogenesis

**FIGURE 2:**
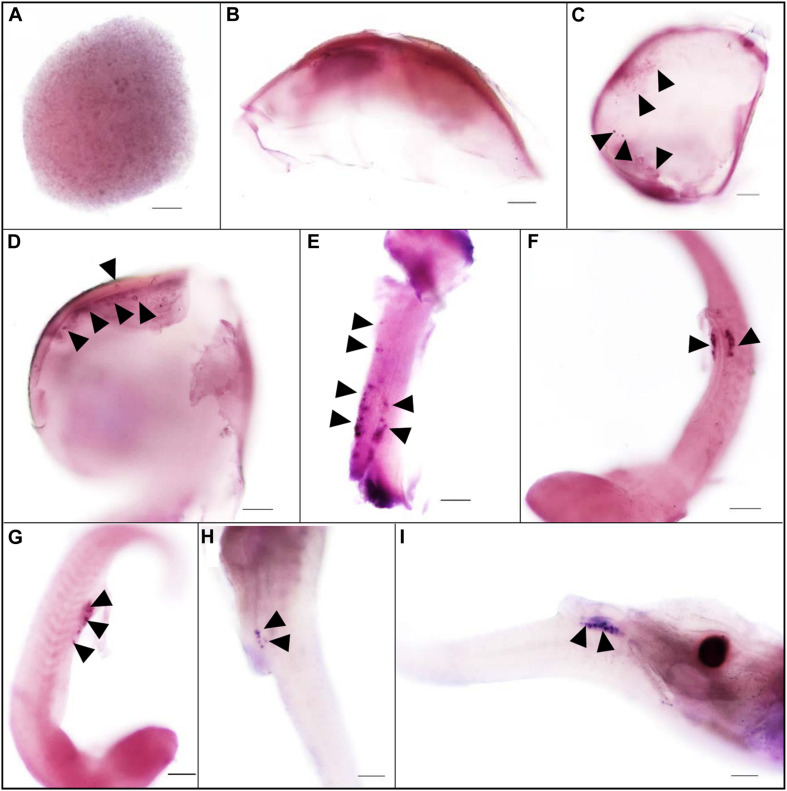
The expression pattern of *Ssvas* during embryogenesis was analyzed with WISH. All blastomeres were strongly stained at the blastula stage **(A)**. The signal in the cell became non-uniform at the 30%-epiboly stage **(B)**. *Ssvas*-positive cells appeared at the 70% epiboly stage **(C)**. PGCs randomly aligned along the anterior of the body axes at the 90% epiboly stage–early neurula stage **(D)** and lined along the anterior–posterior axis on both sides of the embryonic body at the somite stage **(E)**. PGCs formed two clusters and aligned bilaterally on the ventral side at heart beating stage **(F,G)** and localized at the dorsal sides of the gut, ventral side of notochord at hatching Sage **(H,I)**. Black arrowheads indicated *Ssvas*-positive cells (PGCs) (stained with dark purple or blue). Scale bar, 100 μm.

The expression pattern of *vasa* during embryogenesis was analyzed by WISH. At the blastula stage, all blastomeres were strongly stained, without the four cell clusters like zebrafish (Figure 2A). In *Sebastes schlegelii*, the *Ssvas* transcripts failed in early localization during the cleavage stage. As the embryo developed, the distribution of the signal in the cell became non-uniform, and the areas of embryonic shield seemed to be darker than the rest at the 30% epiboly stage (Figure 2B). Until the 70% epiboly stage, some *Ssvas*-positive cells firstly appeared at the germ ring of the epiboly bottom (Figure 2C). According to morphology, these positive cells were early PGCs. At the 90% epiboly stage to early neurula stage, the PGCs gathered on the dorsal side of the narrow embryonic body and randomly aligned along the anterior of the body axes (Figure 2D). Then, they moved posterior-ward and lined along the anterior–posterior axis on both sides of the embryonic body at somite stage (Figure 2E). These cells formed two clusters and aligned bilaterally on the ventral side at heart beating stage (Figures 2F,G). Finally, the PGCs localized at the dorsal sides of the gut, ventral side of notochord at the hatching stage (Figures 2H,I).

### The PGC Migration After Birth of *Sebastes schlegelii*

**FIGURE 3:**
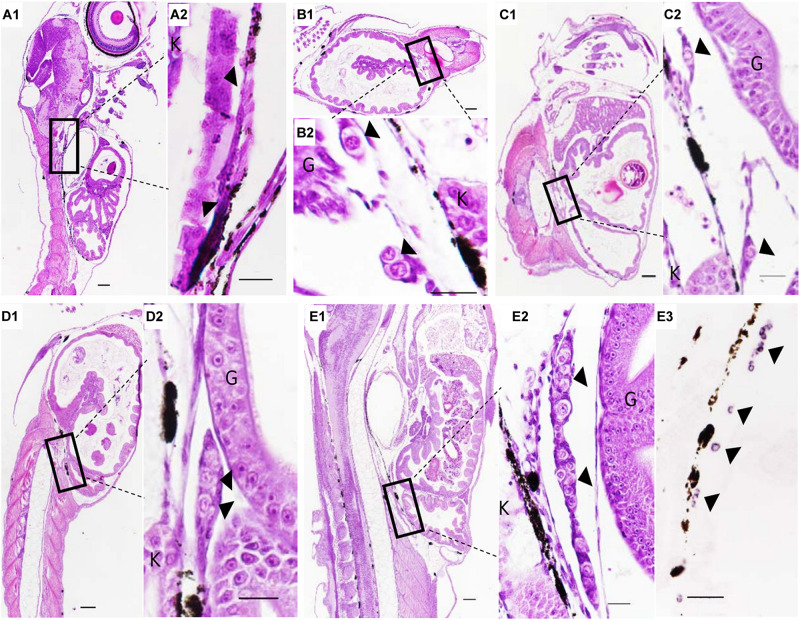
The PGCs migration after birth of *Sebastes schlegelii*. **(A1–E2)** were tissue sections with H&E stain. **(E3)** was SISH corresponding to **(E2)**. PGCs were located in the upper part of the body cavity under the notochord at 2 dab, sagittal sections **(A1,A2)** and covered by a single-layer somatic cell at 8 dab, coronal sections **(B1,B2)**. PGCs migrated to the genital ridge at 9 dab, sagittal sections **(C1,C2)** and formed elongated gonadal primordia at 10 dab, sagittal sections **(D1,D2)**. Distinct primordial gonads were discovered in the posterior part of the body cavity **(E1,E2)**, and the PGCs were strongly stained with the *Ssvas* antisense probe by SISH at 15 dab, sagittal sections **(E3)**. Black arrowheads indicated PGCs; G and K indicated gut and kidney, respectively. Scale bar, 100 μm **(A1,B1,C1,D1,E1)**; 50 μm **(A2,B2,C2,D2,E2,E3)**.

**FIGURE 4:**
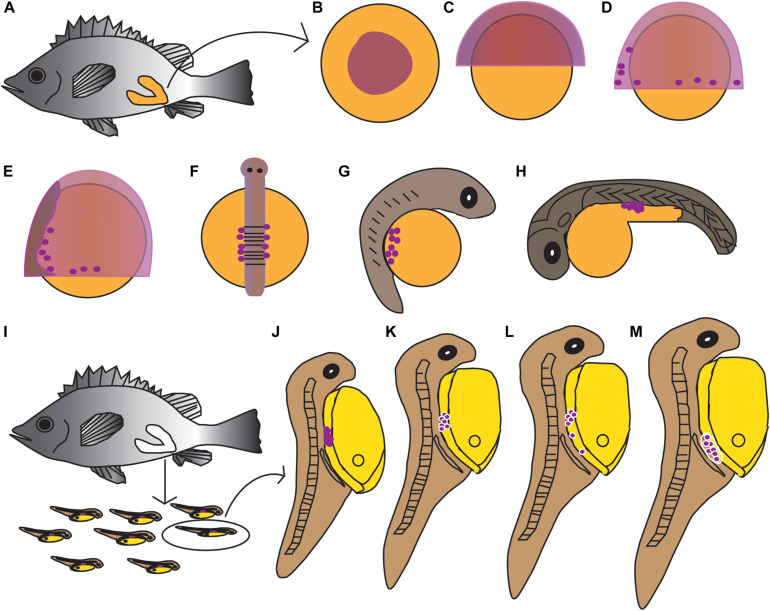
Schematic illustration of the germline formation process in *Sebastes schlegelii*. Dark purple spots indicated PGCs signals. **(A–H)** The migration of PGCs from embryos in female fish after fertilization. **(A)** The overall appearance of the female after fertilization; **(B)** blastula stage; **(C)** 30% epiboly stage; **(D)** 70% epiboly stage; **(E)** 90% epiboly stage–early neurula stage; **(F)** somite stage; **(G)** heart beating stage; **(H)** hatching stage. **(I–M)** The migration of PGCs from larvae after birth. **(I)** The larvae were born from mature females; **(J)** 2 dab; **(K)** 8 dab; **(L)** 9 dab; and **(M)** 10–15 dab.

The PGCs underwent a post-birth migration in *Sebastes schlegelii*. PGCs are characterized by large cell size and obvious nuclei, which are easily distinguished with somatic cells on tissue sections. At 2 days after birth (dab), PGCs localized in the upper part of the body cavity under the notochord, around the dorsal peritoneum (Figures 3A1,A2). A part of PGCs were covered by a single layer somatic cell at 8 dab (Figures 3B1,B2). PGCs surrounded by single somatic cells were found to migrate to the genital ridge at 9 dab (Figures 3C1,C2). By 10 dab, three PGCs were completely surrounded by somatic cells and arranged in a row to form elongated gonadal primordia, which appeared under the ventral kidney and dorsal sides of the gut (Figures 3D1,D2). Until 15 dab, the distinct primordial gonads were discovered in the posterior part of the body cavity, and the PGCs in gonads were strongly stained with *Ssvas* antisense probe by SISH (Figures 3E1–E3). Through the identification of *Ssvas*, the germline formation process of *Sebastes schlegelii* was summarized, as shown in the schematic illustration (Figure 4).

### Localization of *Ssvas* RNA in Gonads

**FIGURE 5:**
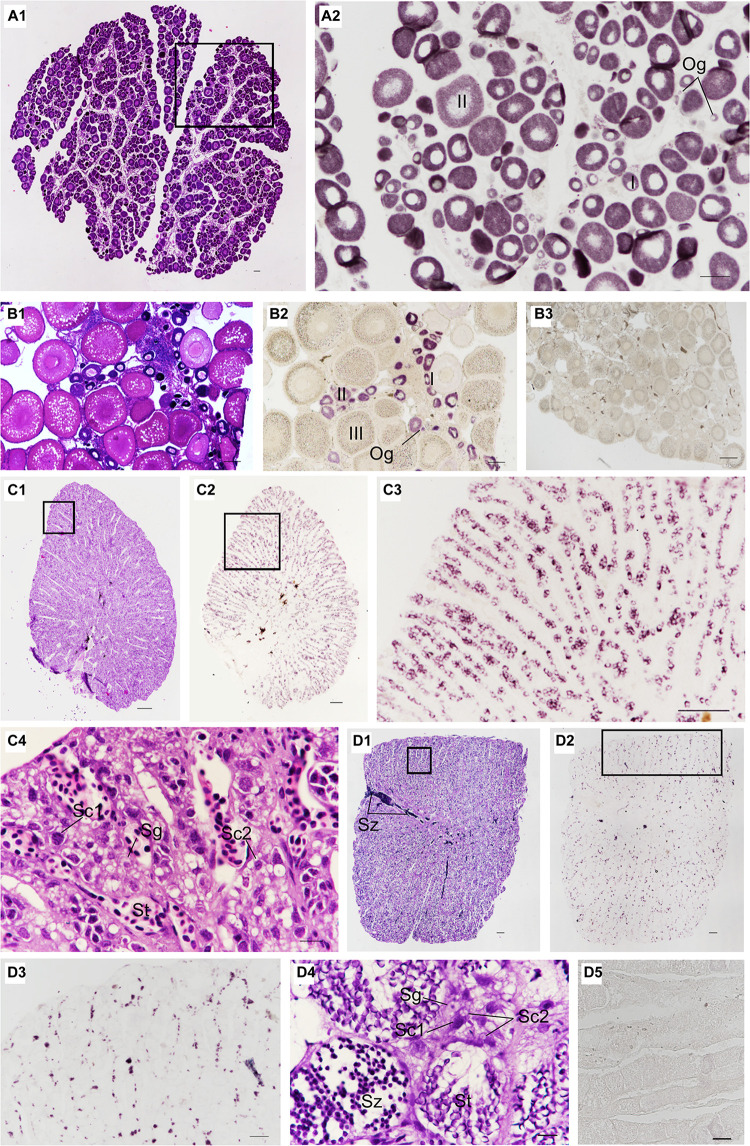
Distribution of *Ssvas* transcripts in gonad germ cells by SISH. Paraffin section and H&E of ovaries in 1-year-old **(A1)** and sexually mature black rockfish **(B1)**, respectively. **(A2)** Adjacent tissue section of the black box in **(A1)** was hybridized with the *Ssvas* antisense probe. **(B2)** Adjacent tissue section of **(B1)** was hybridized with the *Ssvas* antisense probe. **(B3)** The paraffin section of the ovary was hybridized with the *Ssvas* sense probe. The paraffin section and H&E of testis in 1-year-old **(C1)** and sexually mature black rockfish **(D1)**, respectively. **(C2,D2)** Adjacent tissue sections of **(C1,D1)** were hybridized with the *Ssvas* antisense probe, respectively. **(C3,D3)** The high-magnification image of the black boxes in **(C2,D2)**, respectively. **(C4–D4)** The high-magnification image of the black boxes in **(C1,D1)**, respectively. **(D5)** The paraffin section of testis was hybridized with the *Ssvas* sense probe. Og, oogonia; I, early previtellogenic phase; II, late previtellogenic phase; III, early vitellogenic phase; Sg, spermatogonia; Sc1, primary spermatocytes; Sc2, secondary spermatocytes; St, spermatids; Sz, spermatozoon. Scale bar, 100 μm **(A1–C4,D3–D5)**; 200 μm **(D1,D2)**.

The localization of *Ssvas* RNA was investigated in fish gonads by SISH. In accordance with adjacent histology, the stages of stained cells were identified. For 1-year-old black rockfish, in the ovary, there were mainly oogonia, early previtellogenic phase (I), and late previtellogenic phase (II) (Figure 5A1). *Ssvas*-specific expression was observed in all germ cells but not somatic cells (Figure 5A2). In oogonia, *Ssvas* mRNA was distributed evenly in the cytoplasm (Figure 5A2). In I–II oocytes, *Ssvas* transcripts were concentrated into several patches primarily in the perinuclear region (Figure 5A2). For sexually mature females, *Ssvas* transcripts were researched throughout oogenesis and showed a dynamic localization pattern in the ovary. A large number of early vitellogenesis phase (III) oocytes were present in the November individuals, but there was no *Ssvas* expression (Figures 5B1,B2). The tissue section hybridized with the sense probe was not stained, proving that this signal represents the expression of *Ssvas* (Figure 5B3).

Histological sections of the testis demonstrated that *Sebastes schlegelii* was lobular-type (Figures 5C1, D1). In the testis of 1-year-old black rockfish, there were spermatogonia, primary spermatocytes, secondary spermatocytes, and spermatids, without spermatozoon (Figure 5C4). For sexually mature males more than 3 years old, a lot of spermatozoon were found (Figure 5D4). The testis was composed of many testicular lobules, and the *Ssvas* signals were only present in spermatogonia and primary spermatocytes, which localized at the edge of the lobules and spread throughout the whole testis except the vas deferens (Figures 5C2,D2). The signals in matured testis were significantly weaker than 1-year-old testis because *Ssvas* transcripts were predominantly detected in early germ cells, such as spermatogonia and spermatocytes, and no signal was detected in spermatids and spermatozoon (Figures 5C3,D3). As control, the tissue section hybridized with the sense probe was not stained (Figure 5D5).

### Visualization the PGCs of Zebrafish and Marine Medaka by *Sebastes schlegelii vasa* 3′UTR

**FIGURE 6:**
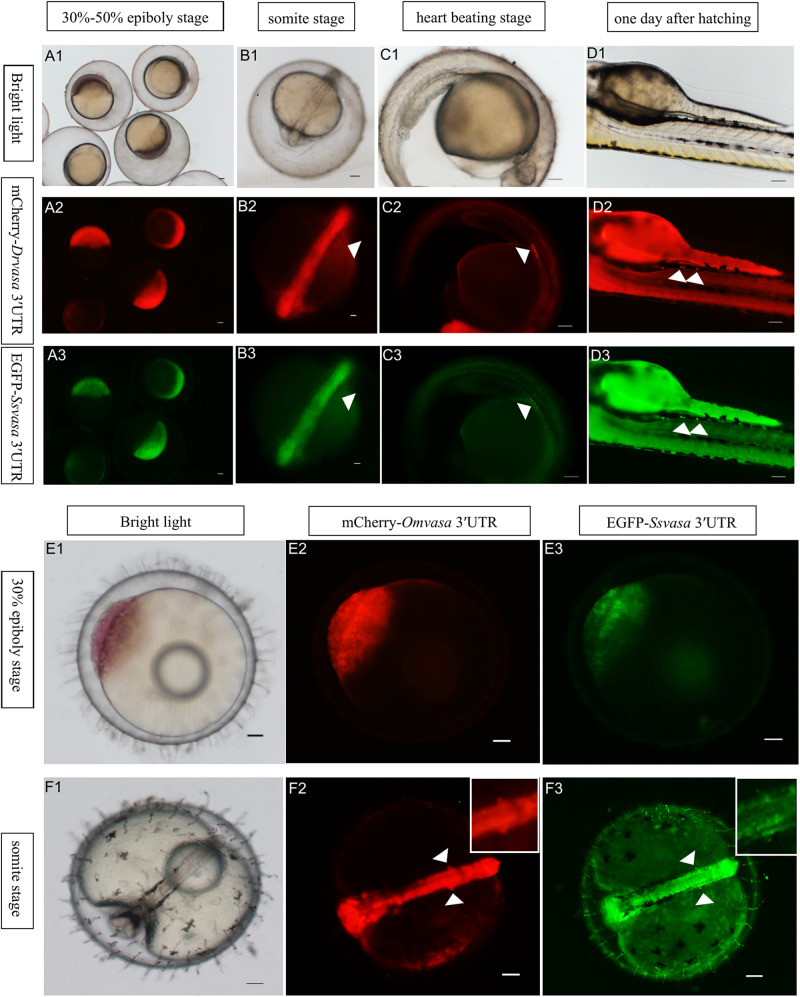
Visualization of the PGCs of zebrafish and marine medaka by *Sebastes schlegelii vasa* 3′UTR. **(A1–D3)** Visualization of zebrafish PGCs by co-microinjection EGFP-*Ssvas* 3′UTR and mCherry-*Drvasa* 3′UTR mRNAs. Fluorescence first expressed in the blastoderm at the 30–50% epiboly stage **(A1–A3)**; PGCs differentiated from somatic cells and gathered on both sides of the embryonic body at the somite stage **(B1–B3)**; PGCs aggregated under the trunk at the heart beating stage **(C1–C3)** and settled on the dorsal part of the gut at 1 day after hatching **(D1–D3)**. **(E1–F3)** Visualization marine medaka PGCs by co-microinjection of EGFP-*Ssvas* 3′UTR and mCherry-*Omvasa* 3′UTR mRNAs. Fluorescence appeared at the 30% epiboly stage **(E1–E3)** and PGCs aligned on both sides of the trunk at the somite stage **(F1–F3)**. The insets in F2 and F3 were magnification of signal areas. PGCs were indicated by white arrows. Scale bars, 100 μm.

In order to confirm that the labeling ability of *Sebastes schlegelii vasa* 3′UTR, EGFP-*Ssvasa* 3′UTR mRNA and mCherry-*Drvasa* 3′UTR mRNA were co-microinjected into zebrafish 1–2 cell stage fertilized eggs. After microinjection, mCherry and EGFP firstly expressed in the blastoderm at the 30–50% epiboly stage, and bright fluorescence accumulated in the area where the embryonic shield formed (Figures 6A1–A3). As embryogenesis proceeded, fluorescence concentrated on the embryonic body was more obvious, but PGCs were not distinguished from somatic cells until the early segmentation period. At the somite stage, as the fluorescence of somatic cells reduced gradually, some brighter cells moved and gathered on both sides of embryonic body (Figures 6B1–B3). The rounder and larger fluorescent cells were PGCs. PGCs migrated axial-ward and aggregated under the trunk at the heart beating stage (Figures 6C1–C3). Eventually, PGCs settled on the dorsal part of the gut at one day after hatching (Figures 6D – D3).

In the same way, the EGFP-*Ssvasa* 3′UTR and mCherry-*Omvasa* 3′UTR mRNAs were co-microinjected into marine medaka embryos. EGFP and mCherry firstly appeared at the 30% epiboly stage (Figures 6E1–E3). At the somite stage, some cells with strong fluorescence aligned on both sides of the trunk from the first somite to tail bud region (Figures 6F1–F3). These fluorescent cells were PGCs.

### *Oryzias melastigma* and *Pagrus major vasa* 3′UTRs Labeling Zebrafish PGCs

**FIGURE 7:**
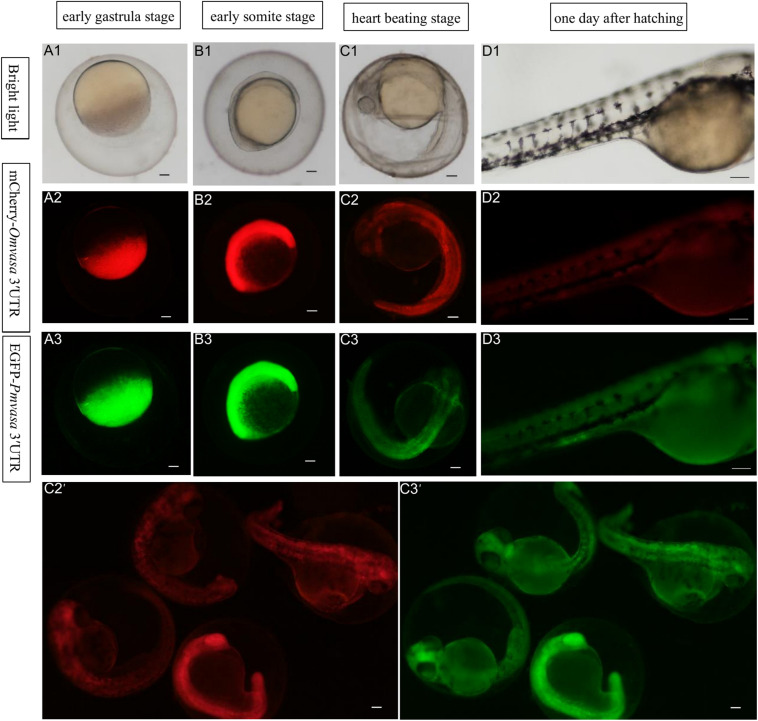
The ability of *Oryzias melastigma* and *Pagrus major vasa* 3′UTRs in labeling zebrafish PGCs. The EGFP*-Pmvasa* 3′UTR and mCherry-*Omvasa* 3′UTR mRNAs were co-microinjection into zebrafish embryos. Fluorescence appeared at the early gastrula stage **(A1–A3)** and highly expressed in the embryonic body at the early somite stage **(B1–B3)**; PGCs did not differentiate from somatic cells at the heart beating stage **(C1–C3)** and 1 day after hatching **(D1–D3)**. View of the embryos of the heart beating stage in a wide range **(C2′,C3′)**. Scale bars, 100 μm.

To compare the labeling ability of *vasa* 3′UTRs among *Sebastes schlegelii* and other teleost in marking zebrafish PGCs, the chimeric mRNAs of EGFP*-Pmvasa* 3′UTR and mCherry-*Omvasa* 3′UTR were also microinjected into zebrafish fertilized eggs. The mCherry and EGFP fluorescence firstly appeared in blastoderm at the early gastrula stage after microinjection (Figures 7A1–A3). A high level of fluorescence expression was found in the zebrafish embryonic body at the early somite stage (Figures 7B1–B3), but PGCs were not distinguished from somatic cells during the whole embryonic development (Figures 7C1–D3). In order to avoid misjudgment of results, a large number of embryos at heart beating stage were observed (Figures 7C2′,C3′). Although some fluorescent aggregates were found in the extension of the yolk sac at heart beating stage and 1 day after hatching, PGCs could not be distinguished (Figures 7C1–C3, D1–D3).

### Phylogenetic Analysis and MEME Motifs Analysis in Teleost *vasa* 3′UTRs

**FIGURE 8:**
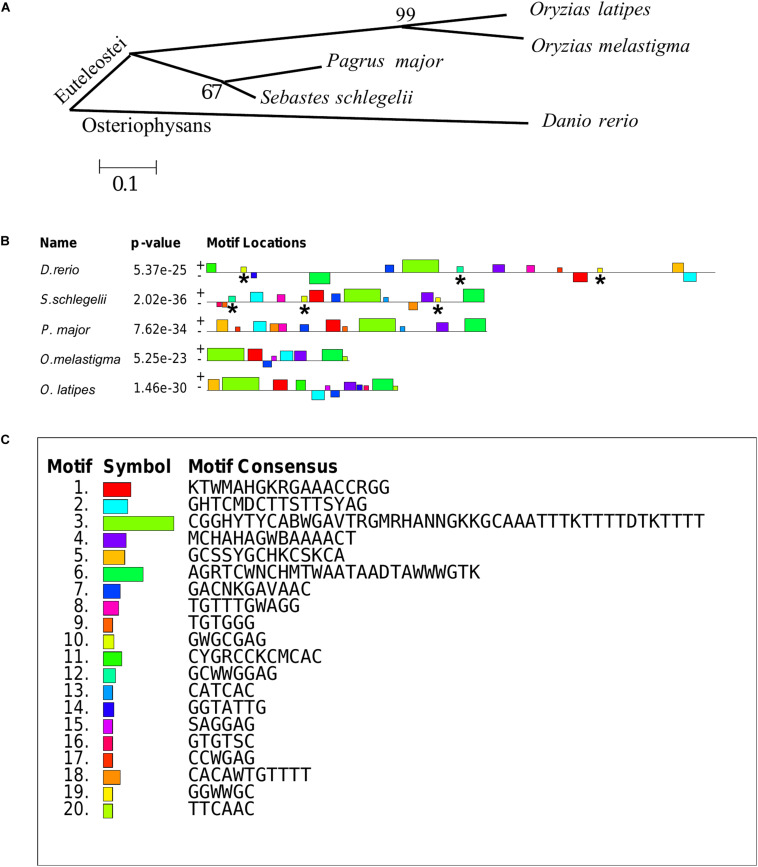
Phylogenetic analysis and MEME motifs analysis in teleost *vasa* 3′UTRs. **(A)** Phylogenetic analysis of teleost *vasa* 3′UTRs by MEGA4. **(B,C)** Conservative functional motifs analysis in teleost *vasa* 3′UTRs by MEME. The twenty motifs designated as M1–M20 were indicated by different color codes **(B)**, and every color code represented motif consensus **(C)**. The motifs of M10, M12, and M19 marked with asterisks were only localized in *Danio rerio* and *Sebastes schlegelii* rather than the other three species.

The teleost of *Sebastes schlegelii*, *Pagrus major*, *Oryzias latipes*, *Oryzias melastigma*, and *Danio rerio* were comparative analysis in this study. Phylogenetic analysis showed that *Sebastes schlegelii* and *Pagrus major* were clustered in one clade, while *Oryzias latipes* and *Oryzias melastigma* were clustered in one clade, both of which belonged to Euteleostei species (Figure 8A). And *Danio rerio* was grouped into a single branch, belonging to Osteriophysans species (Figure 8A).

M1–M20 motifs of *vasa* 3′UTRs were compared among the five teleost species. *Sebastes schlegelii* and *Pagrus major* had more similar conservative elements, while *Oryzias latipes* and *Oryzias melastigma* had more similar motifs (Figures 8B,C). Moreover, *Danio rerio* showed obvious different motifs with above species (Figures 8B,C). The results of MEME analysis were totally consistent with those of the phylogenetic analysis. However, in comparison with other three species, the motifs of M10, M12, and M19 specifically existed in *Sebastes schlegelii* and *Danio rerio* (Figures 8B,C).

## Discussion

The present study reported that the *vasa* identified the germline in *Sebastes schlegelii*. The *Sebastes schlegelii* Vasa protein possessed typical conserved domains of the DEAD-box protein family. By WISH, the *Ssvas* mRNA distributed uniformly throughout each blastomere at the blastula stage, failing in early localization during the cleavage stage. It was not until the late gastrula stage that PGCs gradually formed and randomly aligned along the anterior of the body axes. Then, PGCs moved posterior-ward and lined both sides of the embryonic body at the somite stage, and localized at the dorsal sides of the gut and ventral side of notochord at hatching stage. After birth, PGCs localized in the upper part of the body cavity. They were covered by single-layer somatic cells, migrated to the genital ridge, and formed elongated gonadal primordia in the posterior part of the body cavity at 10 days. The migration routes and final location of PGCs during embryogenesis were similar to medaka (Shinomiya et al., 2000). Moreover, the time of primordial gonad formation of the viviparous economic fish after birth was earlier than most of oviparous fish such as turbot, Japanese flounder, and rainbow trout after hatching, which might be related to the long embryonic development time of viviparous fish *in vivo* (Fernandez et al., 2015; Zhao et al., 2017; Yang et al., 2018). This study firstly described the origination and migration pattern of PGCs in viviparous marine economic fish.

Medaka, rainbow trout, and rainbowfish (*Melanotaenia fluviatilis*) belonged to Euteleostei species, in which the *vasa* transcripts failed to localize at the cleavage furrows (Knaut et al., 2002). However, in the other Euteleostei, including turbot (Lin et al., 2012b), ukigori (Saito et al., 2004) and Atlantic cod (Presslauer et al., 2012), *vasa* mRNA is located at the edge of the first two cleavage furrows, which was similar to that reported in Osteriophysans species, such as zebrafish and carp (*Cyprinus carpio*) (Knaut et al., 2002). We found that the black rockfish was another new viviparous species that lacked the early localization ability in Euteleostei. Although black rockfish, medaka, Atlantic cod, and turbot were closely related in phylogeny, the *vasa* RNA early localization modes were totally different. This indicated that early germ plasm distribution and PGCs origination in different species had no direct relationship with evolution.

It has been proven that the 3′UTR of *vasa* plays a critical role for the stabilization of chimeric RNA in PGCs but not in somatic cells (Knaut et al., 2002; Wolke et al., 2002; Kataoka et al., 2006; Mishima et al., 2006). Visualization fish PGCs *in vivo* could be achieved by chimeric RNA fused fluorescent protein coding sequence to *vasa* 3′UTR (Knaut et al., 2002; Lin et al., 2012a; Ye et al., 2016). This labeling function of *vasa* 3′UTR was relatively conservative, and the PGCs of one species could be labeled by 3′UTRs from highly diverged taxonomic groups (Yoshizaki et al., 2005; Saito et al., 2006; Zhou et al., 2019). For example, PGCs of rainbow trout could be visualized by *vasa* 3′UTRs from both Nibe croaker (*Nibea mitsukurii*) and zebrafish (Yoshizaki et al., 2005). Also, the turbot PGCs could be traced by *vasa* 3′UTR from zebrafish (Zhou et al., 2019). However, the chimeric mRNA of the medaka (GFP-*olvasa* 3′UTR mRNA) failed to label the PGCs in zebrafish or loach (*Misgurnus anguillicaudatus*) embryos (Saito et al., 2006). The previous study proved that zebrafish had a 180-nucleotide element that encompassed the second conserved region of the *vasa* 3′UTR, which was sufficient for *vasa* mRNA localization to the germ plasm, while medaka lacked this region (Knaut et al., 2002). In this study, we firstly discovered that the *vasa* 3′UTR of black rockfish could be used to mark both zebrafish with early localization and medaka without early localization, while the *vasa* 3′UTR of red seabream and marine medaka that lacked early localization (article in publishing) failed to trace the PGCs of zebrafish. These results indicated that the *vasa* 3′UTRs of teleost showed a difference in labeling zebrafish PGCs, which may depend on the specific functional motifs in 3′UTR.

Therefore, we compared the twenty most important motifs of *vasa* 3′UTRs between black rockfish and other four typical fish species. We discovered that, although the black rockfish had the further genetic relationship with zebrafish than other four species in phylogeny, besides some basal motifs similar to the other four specie, there were three motifs of M10, M12, and M19 specifically presented in zebrafish which only existed in black rockfish rather than the other three species. These three specific motifs might partially explain why only the *vasa* 3′UTR of black rockfish was able to successfully trace PGCs in zebrafish. All of the above results also indicated that the *vasa* 3′UTR must possess the essential motifs that play an important role in tracing the PGCs instead of phylogenetic relationships.

In conclusion, we described the expression pattern of *Ssvas* during germ cell origination, development, and maturation process. We firstly reported a viviparous marine economic fish species which failed in early localization until the late gastrula stage. Interestingly, the *vasa* 3′UTR of black rockfish could be used to label PGCs of zebrafish and marine medaka, representing two different typical origination and migration patterns. Compared to other three Euteleostei species, we found that besides some basal motifs, black rockfish had three motifs just presented in zebrafish. Therefore, we speculated that these motifs might play an important role in labeling zebrafish PGCs. The results of this study will improve our understanding of the localization of the germplasm component and the origination of PGCs in teleost and facilitate germ cell manipulation.

## Data Availability Statement

All datasets presented in this study are included in the article/supplementary material.

## Ethics Statement

The animal study was reviewed and approved by the Institutional Animal Care and Use Committee, Institute of Oceanology, Chinese Academy of Sciences.

## Author Contributions

LZ, QL, and JL conceived and designed the study. LZ and SD contributed to the experimental work. LZ and QL contributed to the manuscript writing and revision, respectively. XW, HZ, TD, JY, LW, and ZS contributed with the experimental materials. All authors read and approved the final manuscript.

## Conflict of Interest

ZS was employed by the company Weihai Shenghang Aquatic Product Science and Technology Co., Ltd. The remaining authors declare that the research was conducted in the absence of any commercial or financial relationships that could be construed as a potential conflict of interest.

## Footnotes

1 http://www.ncbi.nlm.nih.gov/
